# Supplementary material for: Adjuvant Use of Pembrolizumab for Stage III Melanoma in a Real-World Setting in Europe
Source: Cancers (Basel). 2024 Oct 22;16(21):3558. doi: 10.3390/cancers16213558 (PMC11545249; doi:10.3390/cancers16213558)
Supplement: Supplementary file 1 [file cancers-16-03558-s001.zip › cancers-3204401-supplementary.pdf]

Supplementary Materials

Table S1: Baseline demographics and clinical characteristics stratified by age

	< 70 (N=136)	>= 70 (N=64)	Overall (N=200)
**AJCC stage (8th edition)**			
Stage IIIA	15 (11.0%)	6 (9.4%)	21 (10.5%)
Stage IIIB	37 (27.2%)	15 (23.4%)	52 (26.0%)
Stage IIIC	81 (59.6%)	39 (60.9%)	120 (60.0%)
Stage IIID	3 (2.2%)	4 (6.3%)	7 (3.5%)
**Gender**			
Female	61 (44.9%)	22 (34.4%)	83 (41.5%)
Male	75 (55.1%)	42 (65.6%)	117 (58.5%)
**BRAF status**			
Positive	53 (39.0%)	21 (32.8%)	74 (37.0%)
Negative	67 (49.3%)	35 (54.7%)	102 (51.0%)
Unknown	16 (11.8%)	8 (12.5%)	24 (12.0%)
**Melanoma subtype**			
Cutaneous Melanoma	132 (97.1%)	58 (90.6%)	190 (95.0%)
MUP	4 (2.9%)	6 (9.4%)	10 (5.0%)
**ECOG**			
0	109 (80.1%)	47 (73.4%)	156 (78.0%)
1	4 (2.9%)	2 (3.1%)	6 (3.0%)
Unknown	23 (16.9%)	15 (23.4%)	38 (19.0%)
**At least one documented comorbidity**			
no	78 (57.4%)	16 (25.0%)	94 (47.0%)
yes	58 (42.6%)	48 (75.0%)	106 (53.0%)

Patient demographics and disease characteristics at start of first pembrolizumab treatment in the adjuvant setting stratified by age. N: number of patients included in the analysis; MUP: Melanoma of unknown primary; AJCC: American Joint Committee on Cancer; BRAF: BRAF mutation status; ECOG: Eastern Cooperative Oncology Group.

Table S2: Baseline demographics and clinical characteristics stratified by BRAF mutation status

	Positive (N=74)	Negative (N=102)	Unknown (N=24)	Overall (N=200)
**AJCC stage (8th edition)**				
Stage IIIA	12 (16.2%)	6 (5.9%)	3 (12.5%)	21 (10.5%)
Stage IIIB	22 (29.7%)	24 (23.5%)	6 (25.0%)	52 (26.0%)
Stage IIIC	39 (52.7%)	67 (65.7%)	14 (58.3%)	120 (60.0%)
Stage IIID	1 (1.4%)	5 (4.9%)	1 (4.2%)	7 (3.5%)
**Gender**				
Female	35 (47.3%)	38 (37.3%)	10 (41.7%)	83 (41.5%)
Male	39 (52.7%)	64 (62.7%)	14 (58.3%)	117 (58.5%)
**Age (years)**				
Mean (SD)	56.4 (16.2)	63.0 (13.4)	60.6 (15.3)	60.3 (15.0)
Median [Min, Max]	57.5 [22.0, 88.0]	64.0 [28.0, 85.0]	64.0 [19.0, 82.0]	63.0 [19.0, 88.0]
**Age (years)**				
< 70	53 (71.6%)	67 (65.7%)	16 (66.7%)	136 (68.0%)
≥ 70	21 (28.4%)	35 (34.3%)	8 (33.3%)	64 (32.0%)
**Melanoma subtype**				
Cutaneous Melanoma	71 (95.9%)	95 (93.1%)	24 (100%)	190 (95.0%)
MUP	3 (4.1%)	7 (6.9%)	0 (0%)	10 (5.0%)
**ECOG**				
0	63 (85.1%)	75 (73.5%)	18 (75.0%)	156 (78.0%)
1	2 (2.7%)	4 (3.9%)	0 (0%)	6 (3.0%)
Unknown	9 (12.2%)	23 (22.5%)	6 (25.0%)	38 (19.0%)
**At least one documented comorbidity**				
no	36 (48.6%)	47 (46.1%)	11 (45.8%)	94 (47.0%)
yes	38 (51.4%)	55 (53.9%)	13 (54.2%)	106 (53.0%)

Patient demographics and disease characteristics at start of first pembrolizumab treatment in the adjuvant setting stratified by BRAF mutation status. N: number of patients included in the analysis; SD: Standard deviation; Min: minimum; Max: maximum; MUP: Melanoma of unknown primary; AJCC: American Joint Committee on Cancer; BRAF: BRAF mutation status; ECOG: Eastern Cooperative Oncology Group.

Table S3: Baseline demographics and clinical characteristics stratified by AJCC stage (8th edition)

	Stage IIIA (N=21)	Stage IIIB (N=52)	Stage IIIC (N=120)	Stage IIID (N=7)	Overall (N=200)
**Gender**					
Female	12 (57.1%)	22 (42.3%)	47 (39.2%)	2 (28.6%)	83 (41.5%)
Male	9 (42.9%)	30 (57.7%)	73 (60.8%)	5 (71.4%)	117 (58.5%)
**Age (years)**					
Mean (SD)	55.5 (17.3)	59.3 (14.3)	60.7 (14.8)	73.7 (7.36)	60.3 (15.0)
Median [Min, Max]	60.0 [19.0, 77.0]	59.0 [29.0, 84.0]	63.5 [22.0, 88.0]	76.0 [64.0, 82.0]	63.0 [19.0, 88.0]
**Age (years)**					
< 70	15 (71.4%)	37 (71.2%)	81 (67.5%)	3 (42.9%)	136 (68.0%)
≥ 70	6 (28.6%)	15 (28.8%)	39 (32.5%)	4 (57.1%)	64 (32.0%)
**BRAF**					
Negative	6 (28.6%)	24 (46.2%)	67 (55.8%)	5 (71.4%)	102 (51.0%)
Positive	12 (57.1%)	22 (42.3%)	39 (32.5%)	1 (14.3%)	74 (37.0%)
Unknown	3 (14.3%)	6 (11.5%)	14 (11.7%)	1 (14.3%)	24 (12.0%)
**Melanoma subtype**					
Cutaneous Melanoma	21 (100%)	48 (92.3%)	114 (95.0%)	7 (100%)	190 (95.0%)
MUP	0 (0%)	4 (7.7%)	6 (5.0%)	0 (0%)	10 (5.0%)
**ECOG**					
0	14 (66.7%)	41 (78.8%)	95 (79.2%)	6 (85.7%)	156 (78.0%)
1	1 (4.8%)	2 (3.8%)	3 (2.5%)	0 (0%)	6 (3.0%)
Unknown	6 (28.6%)	9 (17.3%)	22 (18.3%)	1 (14.3%)	38 (19.0%)
**At least one documented comorbidity**					
no	12 (57.1%)	20 (38.5%)	58 (48.3%)	4 (57.1%)	94 (47.0%)
yes	9 (42.9%)	32 (61.5%)	62 (51.7%)	3 (42.9%)	106 (53.0%)

Patient demographics and disease characteristics at start of first pembrolizumab treatment in the adjuvant setting stratified by AJCC stage. N: number of patients included in the analysis; SD: Standard deviation; Min: minimum; Max: maximum; MUP: Melanoma of unknown primary; AJCC: American Joint Committee on Cancer; BRAF: BRAF mutation status; ECOG: Eastern Cooperative Oncology Group.

Table S4: Time on treatment by demographic and disease characteristics

	Events, n (%)	Median TOT months, (95% CI)	Q1, Q3
**Age**			
< 70	116 (85.3%)	11.1 (9.9-11.7)	5.3, 12
≥ 70	50 (78.1%)	9.6 (5.4-11.8)	2.1, 12
**BRAF**			
Positive	66 (89.2%)	9 (6.4-11.3)	3.5, 12
Negative	81 (79.4%)	11.1 (9.2-11.8)	3.9, 12
**Gender**			
Female	69 (83.1%)	11.1 (9.6-11.8)	5.5, 12
Male	97 (82.9%)	9.9 (6.9-11.6)	3.1, 11.9
**AJCC Stage (8th edition)**			
Stage IIIA	16 (76.2%)	11.8 (11.1-12.9)	9, 12.7
Stage IIIB	46 (88.5%)	11.1 (9.9-11.8)	3.3, 11.8
Stage IIIC	98 (81.7%)	9.4 (6.9-11.6)	3.5, 12
Stage IIID*	6 (85.7%)	12 (2.8-NR)	2.8, 12.2

N: Number of patients included in the analysis, TOT: time on treatment; AJCC: American Joint Committee on Cancer; BRAF: BRAF mutation status; CI: Confidence interval; NR: not reached. * Only seven patients were included in this group, therefore estimates are uncertain.

Table S5: Recurrence-free survival by demographic and disease characteristics

	Events, n (%)	Median RFS Months, (95% CI)	Q1, Q3
**Age**			
< 70	53 (39%)	24.3 (18.2- NR)	7.2, NR
≥ 70	22 (34.4%)	NR (15.7- NR)	6, NR
**BRAF**			
Positive	34 (45.9%)	22.3 (11.8- NR)	6, NR
Negative	37 (36.3%)	29.6 (16.2- NR)	6.6, NR
**Gender**			
Female	29 (34.9%)	NR (16.2- NR)	12, NR
Male	46 (39.3%)	29.6 (18.2- NR)	5.8, NR
**AJCC Stage (8th edition)**			
Stage IIIA	4 (19%)	NR (29.6- NR)	29.6, NR
Stage IIIB	16 (30.8%)	NR (19.8- NR)	12.4, NR
Stage IIIC	53 (44.2%)	18.7 (12.5- NR)	6, NR
Stage IIID*	2 (28.6%)	NR (2.9- NR)	2.9, NR

N: Number of patients included in the analysis, RFS: recurrence-free survival; AJCC: American Joint Committee on Cancer; BRAF: BRAF mutation status; CI: Confidence interval; NR: not reached. *Only seven patients were included in this group, therefore estimates are uncertain.

Table S6: Distant metastasis-free survival by demographic and disease characteristics

	Events, n (%)	Median DMFS Months, (95% CI)	Q1, Q3
**Age**			
< 70	39 (28.7%)	NR (22.7-NR)	15.6, NR
≥ 70	23 (35.9%)	32.4 (18.6-NR)	11.9, 32.4
**BRAF**			
Positive	31 (41.9%)	21.7 (19.2-NR)	12, NR
Negative	28 (27.5%)	NR (21.4-NR)	16.6, NR
**Gender**			
Female	23 (27.7%)	32.4 (32.4-NR)	15.6, NR
Male	39 (33.3%)	29.6 (20.2-NR)	14.8, NR
**AJCC Stage (8th edition)**			
Stage IIIA	4 (19%)	NR (29.6-NR)	29.6, NR
Stage IIIB	14 (26.9%)	NR (19.8-NR)	18.4, NR
Stage IIIC	43 (35.8%)	25.6 (20.2-NR)	13.6, NR
Stage IIID*	1 (14.3%)	NR (NR-NR)	NR, NR

N: Number of patients included in the analysis, DMFS: distant metastasis-free survival; RFS: recurrence-free survival; AJCC: American Joint Committee on Cancer; BRAF: BRAF mutation status; CI: Confidence interval; NR: not reached. *Only seven patients were included in this group, therefore estimates are uncertain.

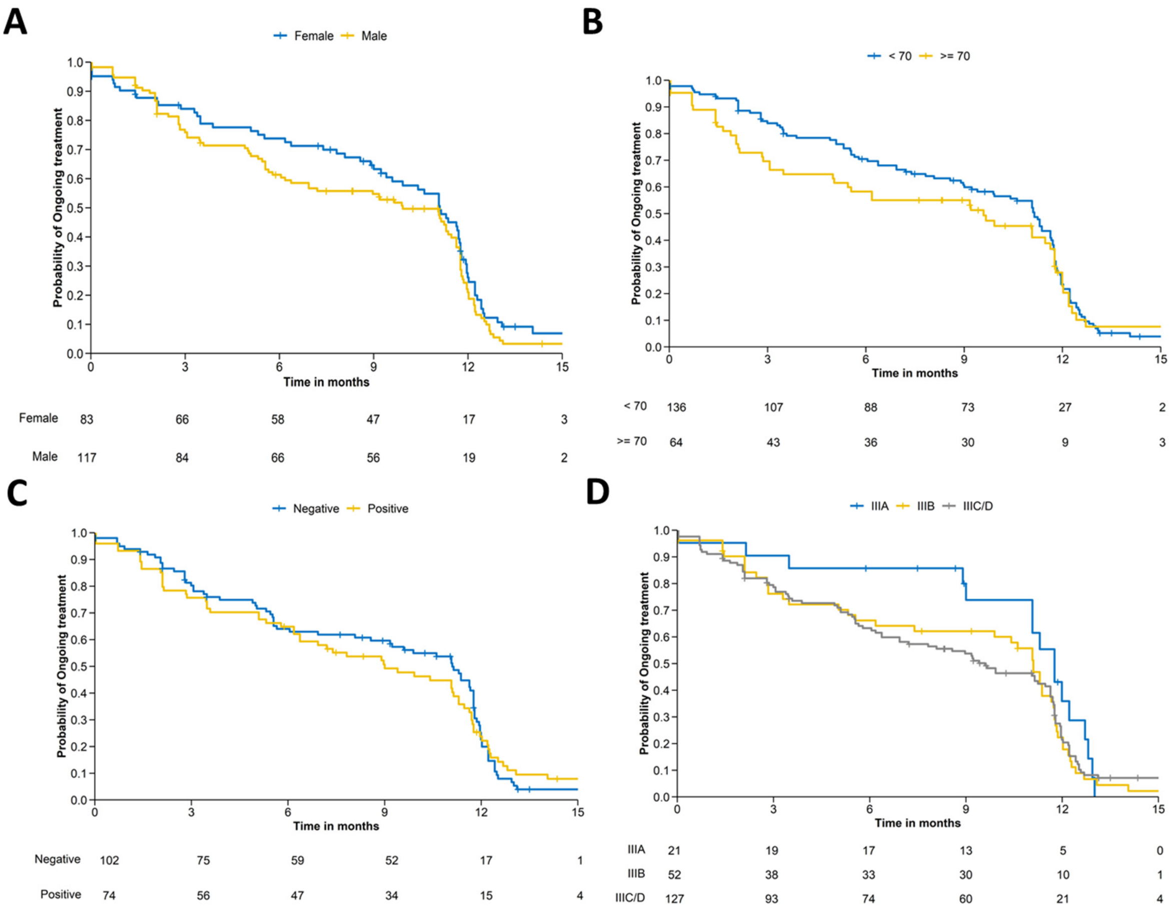

Figure S1. Time on adjuvant pembrolizumab treatment stratified by (A) gender, (B) age, (C) BRAF mutation status, and (D) stage.

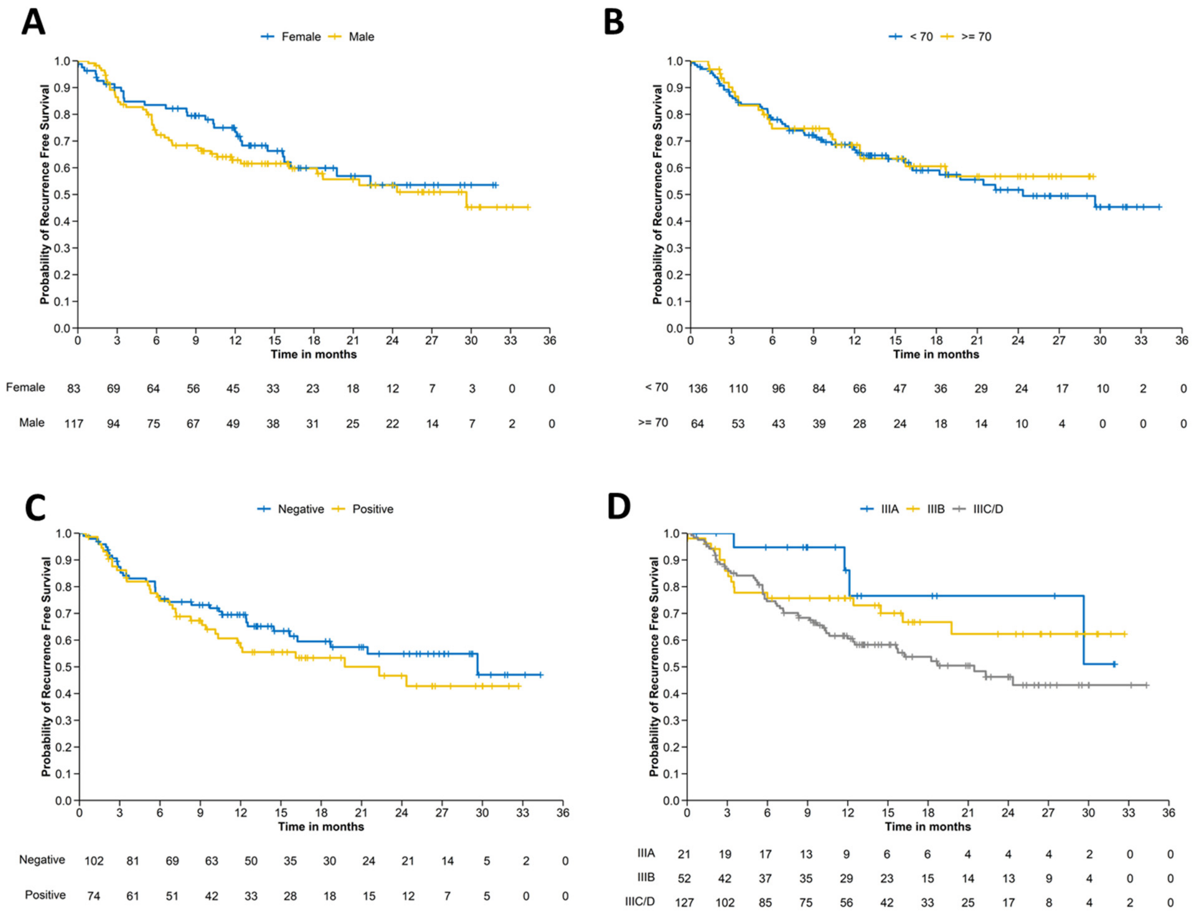

Figure S2. Recurrence-free survival stratified by (A) gender, (B) age, (C) BRAF mutation status, and (D) stage.

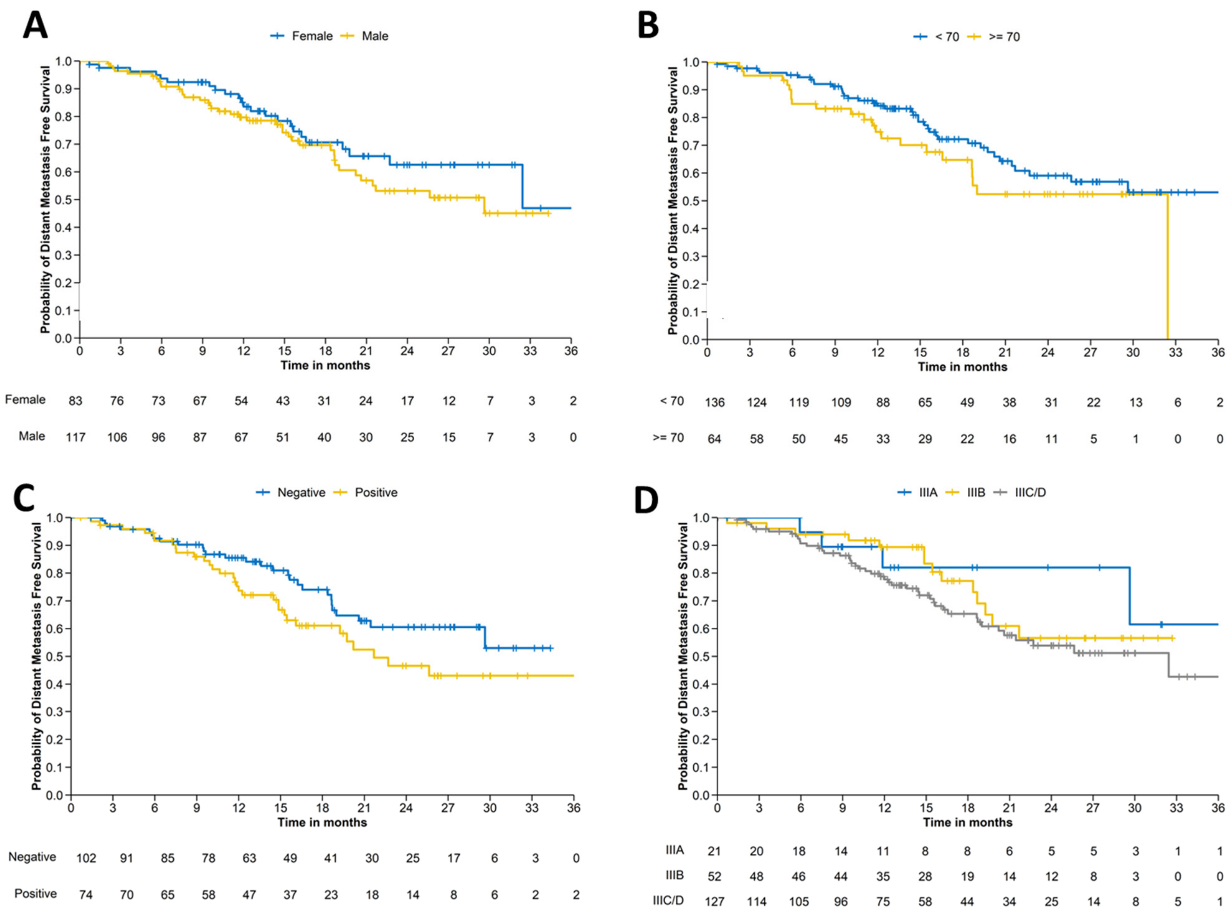

Figure S3: Kaplan-Meier curves of DMFS stratified by (A) age, (B) gender, (C) BRAF mutation status, and (D) stage.

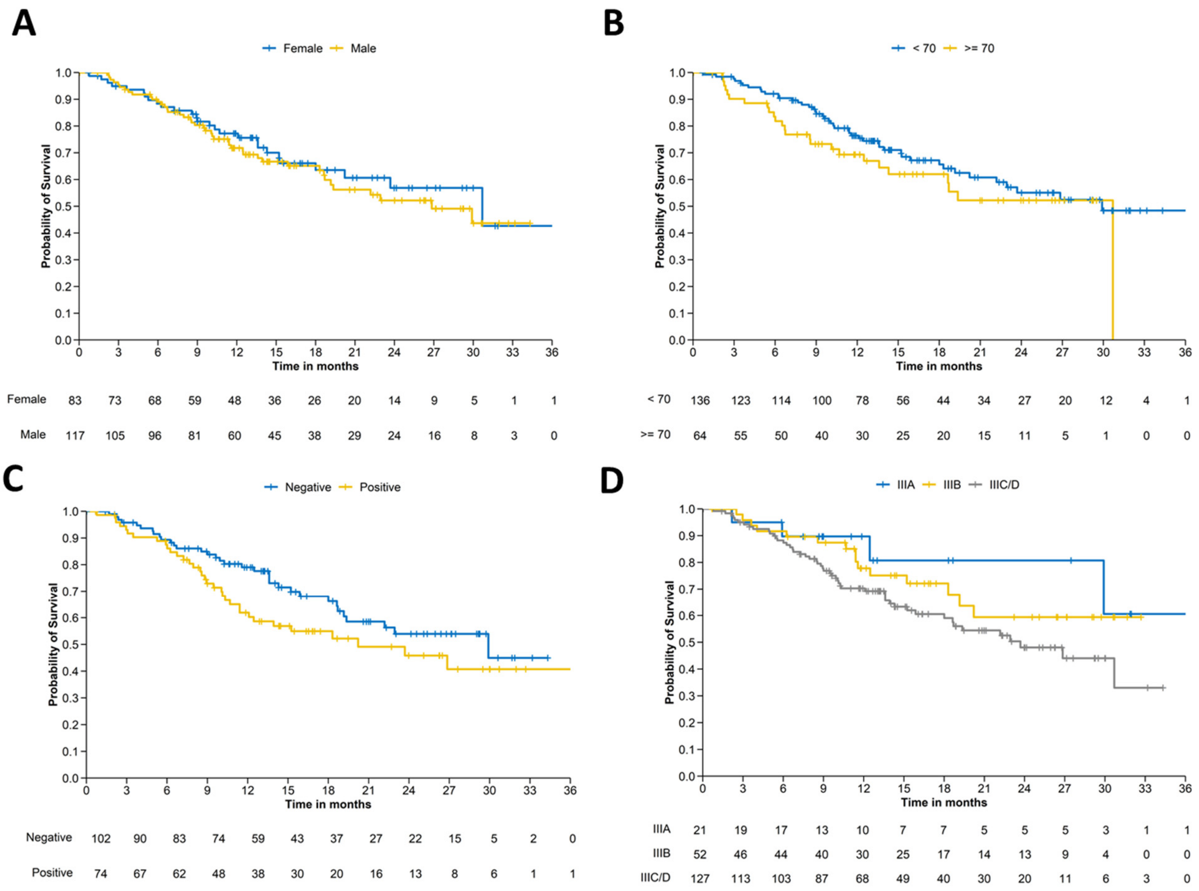

Figure S4: Kaplan-Meier curves of TTNT stratified by (A) age, (B) gender, (C) BRAF mutation status, and (D) stage.

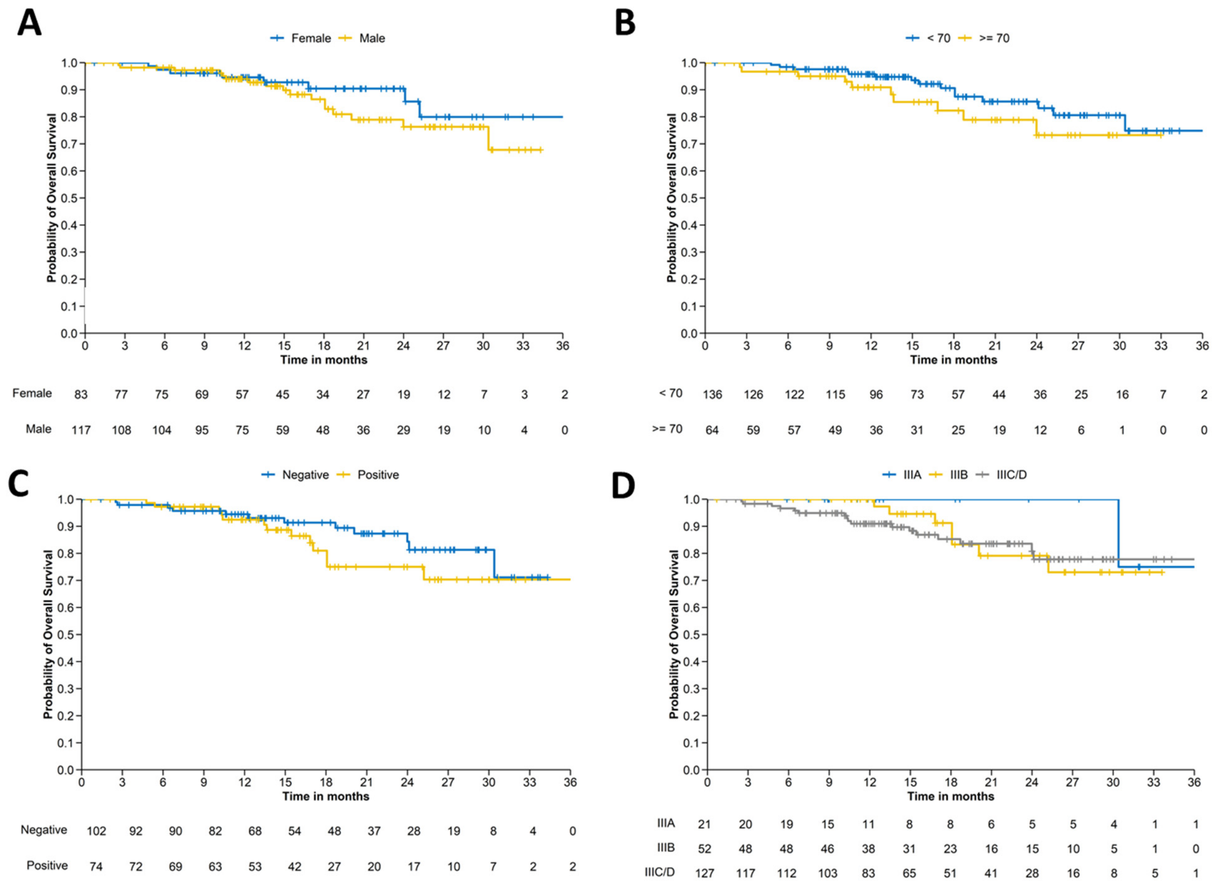

Figure S5: Kaplan-Meier curves of OS stratified by (A) age, (B) gender, (C) BRAF mutation status, and (D) stage.

Table S7: Survival endpoints stratified by age

	< 70 years (N = 136)	≥ 70 years (N = 64)	Total (N = 200)
**12-month survival rate (95% CI)**			
OS	95.8 (92.2-99.5)	90.9 (83.6-98.9)	94.3 (90.8-97.8)
RFS	66.7 (58.8-75.6)	68.5 (57.4-81.9)	67.3 (60.7-74.6)
DMFS	84.2 (77.9-91)	74.8 (64.1-87.3)	81.3 (75.7-87.3)
TTNT	76.4 (69.1-84.5)	69.3 (58.4-82.3)	74.1 (67.9-80.9)
**18-month survival rate (95% CI)**			
OS	90.6 (84.8-96.9)	82.3 (71.5-94.8)	88.1 (82.7-93.8)
RFS	59 (50.3-69.3)	60.6 (48.3-76)	59.6 (52.3-67.9)
DMFS	72.2 (63.8-81.7)	64.7 (52.5-79.9)	70 (62.9-77.8)
TTNT	65.6 (56.9-75.7)	62 (50.1-76.7)	64.5 (57.3-72.6)
**24-month survival rate (95% CI)**			
OS	85.6 (78.1-93.9)	73.2 (58.8-91.2)	81.9 (74.9-89.7)
RFS	51.7 (42.1-63.6)	56.8 (43.8-73.6)	53.4 (45.4-62.8)
DMFS	59.1 (49-71.2)	52.4 (39-70.4)	57.1 (48.8-66.8)
TTNT	55.1 (45-67.3)	52.2 (39.1-69.7)	54.1 (45.9-63.9)

N: number of patients; DMFS: Distant metastasis-free survival. OS: Overall survival, RFS: Relapse-free survival, TTNT: Time to next treatment; CI: Confidence interval.

Table S8: Survival endpoints stratified by gender

	Female (N = 83)	Male (N = 117)	Total (N = 200)
**12-month survival rate (95% CI)**			
OS	94.6 (89.6-99.9)	94 (89.4-98.8)	94.3 (90.8-97.8)
RFS	73.4 (63.9-84.2)	62.9 (54.2-72.9)	67.3 (60.7-74.6)
DMFS	83.5 (75.4-92.6)	79.7 (72.3-87.9)	81.3 (75.7-87.3)
TTNT	77.2 (68.2-87.4)	71.8 (63.5-81.1)	74.1 (67.9-80.9)
**18-month survival rate (95% CI)**			
OS	90.4 (83.2-98.3)	86.5 (79.1-94.5)	88.1 (82.7-93.8)
RFS	59.9 (48.8-73.6)	59.8 (50.7-70.5)	59.6 (52.3-67.9)
DMFS	70.7 (60.1-83.1)	69.6 (60.5-80.1)	70 (62.9-77.8)
TTNT	63.5 (52.4-77)	65.1 (56.1-75.6)	64.5 (57.3-72.6)
**24-month survival rate (95% CI)**			
OS	90.4 (83.2-98.3)	76.3 (66.1-88)	81.9 (74.9-89.7)
RFS	53.6 (41.4-69.3)	53.5 (43.4-65.8)	53.4 (45.4-62.8)
DMFS	62.6 (50.6-77.4)	53.1 (42.4-66.6)	57.1 (48.8-66.8)
TTNT	56.9 (44.4-72.8)	52.2 (41.8-65.2)	54.1 (45.9-63.9)

N: number of patients; DMFS: Distant metastasis-free survival. OS: Overall survival, RFS: Relapse-free survival, TTNT: Time to next treatment; CI: Confidence interval.

Table S9: Survival endpoints stratified by BRAF mutation status

	Positive (N=74)	Negative (N=102)	Total (N = 200)
**12-month survival rate (95% CI)**			
OS	92.4 (86.3-99.1)	94.5 (89.8-99.3)	94.3 (90.8-97.8)
RFS	57.2 (46.4-70.5)	69.5 (60.7-79.7)	67.3 (60.7-74.6)
DMFS	73.7 (63.9-84.9)	85.5 (78.5-93.1)	81.3 (75.7-87.3)
TTNT	61.9 (51.3-74.7)	79 (71-87.9)	74.1 (67.9-80.9)
**18-month survival rate (95% CI)**			
OS	81 (70.7-92.9)	91.4 (85.3-97.8)	88.1 (82.7-93.8)
RFS	53.4 (42.4-67.2)	59.5 (49.4-71.7)	59.6 (52.3-67.9)
DMFS	61.1 (50-74.6)	74 (64.5-84.9)	70 (62.9-77.8)
TTNT	54.9 (44-68.6)	66.2 (56.6-78)	64.5 (57.3-72.6)
**24-month survival rate (95% CI)**			
OS	75 (63.1-89.2)	84.3 (75.1-94.6)	81.9 (74.9-89.7)
RFS	46.7 (34.7-62.8)	54.9 (44.2-68.2)	53.4 (45.4-62.8)
DMFS	46.6 (34.2-63.6)	60.6 (49.4-74.2)	57.1 (48.8-66.8)
TTNT	45.8 (33.9-62)	53.9 (42.7-68.1)	54.1 (45.9-63.9)

N: number of patients; DMFS: Distant metastasis-free survival. OS: Overall survival, RFS: Relapse-free survival, TTNT: Time to next treatment; CI: Confidence interval.

Table S10: Survival endpoints stratified by AJCC stage (8th edition)

	Stage IIIA (N = 21)	Stage IIIB (N = 52)	Stage IIIC (N = 120)	Stage IIID (N = 7)	Total (N = 200)
**12-month survival rate (95% CI)**					
OS	100 (100-100)	100 (100-100)	91.5 (86.3-97)	83.3 (58.3-100)	94.3 (90.8-97.8)
RFS	86.1 (69.5-100)	75.7 (64.6-88.7)	60.1 (51.5-70.2)	66.7 (37.9-100)	67.3 (60.7-74.6)
DMFS	82 (65.2-100)	89.4 (80.9-98.7)	77.4 (69.8-85.8)	83.3 (58.3-100)	81.3 (75.7-87.3)
TTNT	89.7 (77.2-100)	77.8 (66.5-91)	69.5 (61.3-78.8)	83.3 (58.3-100)	74.1 (67.9-80.9)
**18-month survival rate (95% CI)**					
OS	100 (100-100)	91.2 (82.1-100)	85.5 (78.2-93.4)	83.3 (58.3-100)	88.1 (82.7-93.8)
RFS	76.6 (55.8-100)	66.7 (53.9-82.6)	53 (43.8-64.2)	66.7 (37.9-100)	59.6 (52.3-67.9)
DMFS	82 (65.2-100)	77.2 (64.8-91.9)	64.4 (55.1-75.2)	83.3 (58.3-100)	70 (62.9-77.8)
TTNT	80.7 (62.5-100)	72.1 (59.7-87.1)	57.7 (48.3-68.8)	83.3 (58.3-100)	64.5 (57.3-72.6)
**24-month survival rate (95% CI)**					
OS	100 (100-100)	79.1 (65.3-95.9)	80.8 (71.7-91.1)	83.3 (58.3-100)	81.9 (74.9-89.7)
RFS	76.6 (55.8-100)	62.3 (48.4-80.2)	45.3 (35.4-58)	66.7 (37.9-100)	53.4 (45.4-62.8)
DMFS	82 (65.2-100)	56.6 (40.9-78.3)	52.5 (42.2-65.3)	83.3 (58.3-100)	57.1 (48.8-66.8)
TTNT	80.7 (62.5-100)	59.4 (44.4-79.4)	46.3 (36-59.6)	83.3 (58.3-100)	54.1 (45.9-63.9)

N: number of patients; DMFS: Distant metastasis-free survival. OS: Overall survival, RFS: Relapse-free survival, TTNT: Time to next treatment; CI: Confidence interval.
